# Outer Macular Microvascular Supply in Retinitis Pigmentosa Examined using Optical Coherence Tomography Angiography

**DOI:** 10.1155/2021/5575851

**Published:** 2021-12-21

**Authors:** Mingsheng Hong, Jiquan Wen, Jixian Lou, Jiehui Xu, Tingjun Xu

**Affiliations:** ^1^Department of Ophthalmology, Zhejiang Hospital, Hangzhou 310013, China; ^2^Department of Ophthalmology, The First People's Hospital of PingHu, PingHu 314200, China

## Abstract

**Purpose:**

To determine the vessel density of the superior (SCP) and deep retinal capillary plexuses (DCP) in patients with retinitis pigmentosa (RP) using optical coherence tomography angiography (OCTA).

**Methods:**

This was a cross-sectional study. A total of 25 eyes of 25 healthy volunteers and 30 eyes of 17 patients with RP were evaluated in this study. The integrity of the ellipsoid zone in the macular fovea was evaluated as an intact or defect using a spectral-domain OCT. Commercial spectral domain coherence tomography angiography (OCTA) was used to scan the macular region of approximately 3 × 3 mm^2^. The vessel density in the SCP and DCP were calculated after appropriate layer segmentation and removal of projection artifacts. The central retinal thickness (CRT) was measured with automated software. The vessel densities in the SCP and DCP were compared between different groups using SPSS.

**Results:**

A total of 25 eyes of 25 healthy subjects and 30 eyes of 17 patients with RP were evaluated in the study. There was no significant difference in ages between the two groups (*F* = 0.065 and *P*=0.937). There was a significant difference in SCP and DCP between the patients with RP and healthy individuals (*P* < 0.001 and *P* < 0.001). The DCP was significantly reduced in the parafovea region between the macular intact and defect groups (*P* < 0.05), except in the fovea and nasal regions. After linear regression, the DCP/SCP ratio in the whole, fovea, and parafovea regions was closely related to the DCP vessel density (*P* < 0.05), and CRT in the fovea and parafovea was not related to the whole DCP (*P*=0.186 and *P*=0.539).

**Conclusion:**

The vessel density decreased in patients with RP, especially in the DCP of the parafovea region. A greater loss of capillaries in the DCP was found when the macular region was involved. The DCP/SCP ratio may be an important indicator of RP.

## 1. Introduction

Retinitis pigmentosa (RP) is a wide group of hereditary retinal diseases with a prevalence of 1 in 1000–5000 individuals [1]. More than 1 million individuals are affected by the disease [2]. Degeneration of the rods and cones is the main pathological change. RP is one of the leading causes of blindness in elderly individuals aged <60 years [3]. The onset is always the constriction of the peripheral visual field and the ultimate loss of central vision [2]. Mutation in rhodopsin has been identified in patients with RP and is regarded as the key mechanism in photoreceptor degeneration [4]. Moreover, retinal and choroid hemodynamics have been found to decrease with impaired retinal vascular oxygen saturation [5]. Retinal vascular stenosis is a typical manifestation in RP. Therefore, insufficient vascular supply is also another factor in the pathology of RP.

Recent advancements in the noninvasive measurement of retinal blood supply offer new insights into the pathogenesis of patients with RP [6–8]. A previous study found a significant reduction in vessel density (VD) in the whole retinal and perifoveal regions [6, 9]. However, it did not demonstrate a dynamic change in the disease progression. Another study reported that the retinal vascular supply was strongly reduced in the rat model of RP, especially in the outer retinal structure; however, it did not illustrate the vascular condition when the macular region was involved [10]. The macular dysfunction always occurred at a late stage, and the visual acuity (VA) was always poor in patients with RP [11]. However, whether the VD decreased with macular involvement was unknown. Optical coherence tomography angiography (OCTA) has been used to measure the retinal VD to reflect the vascular changes [12]. This study aimed to characterize the alternation of retinal VD in patients with RP and to determine whether the reduction in retinal VD was accompanied by macular dysfunction. The present study indicated that the vessel density is decreased in those with a disrupted ellipsoid zone.

## 2. Materials and Methods

This was a cross-sectional study. Healthy volunteers and patients with RP were recruited from Zhejiang Hospital. Patients with systemic diseases (hypertension, diabetes, and Alzheimer's disease) and other ocular diseases (macular hole, epiretinal membrane, retinal vessel occlusion, glaucoma, age-related macular degeneration, and optic diseases) and patients who previously underwent any ocular surgery were excluded in the study. Written informed consent was provided by the participants. All the participants underwent comprehensive ophthalmologic examinations, including medical history, best-corrected VA measurement using a Snellen chart (VA was changed to logMAR for analysis), slit-lamp examination, color fundus photography, fundus autofluorescence (FA) imaging using the TOPCON camera (Topcon Corporation, IMAGEnet 200, Tokyo, Japan), SD-OCT of the macular region (Heidelberg), and OCTA using Avanti RTVue-XR platform (Optovue, Fremont, CA, USA).

The diagnosis of RP was finally verified using the full-field electroretinogram by the RETIport system (Roland Consult, Brandenburg, Germany). The test was conducted according to the standards of the International Society for Clinical Electrophysiology of Vision.

### 2.1. OCTA Image Acquisition and Analysis

**Figure 1:**
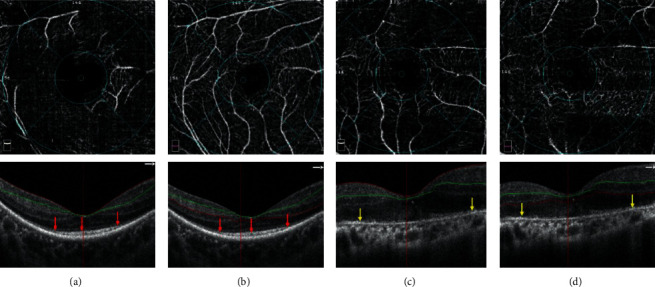
Measurement of vessel density in patients with RP. (a) Superior capillary plexus (SCP) of one patient with RP: the ellipsoid zone is intact. (b) Deep capillary plexus (DCP) of one patient with RP: the ellipsoid zone is intact in the macular fovea. Red arrowhead indicates the ellipsoid zone. (c) SCP of one patient with RP: the ellipsoid zone is broken. (d) DCP of one patient with RP: the ellipsoid zone is broken. Yellow arrowhead indicates the ellipsoid zone.

All the subjects underwent OCTA on the macular 3 × 3 mm^2^ region. The Avanti RTVue-XR system (Optovue) adopted a split-spectrum amplitude-decorrelation angiography algorithm to extract the OCTA information. The transverse resolution was 5 *μ*m and 22 *μ*m in the tissues, respectively. The system has a repetition frequency of 70 k A-scans per second. Each scan consisted of volumetric scans with orthogonal directions (*X*-fast and *Y*-fast). Each volume of OCTA consisted of 304 × 304 A-scans. Projection artifacts were removed by the projection-resolved algorithm as previously conducted [13]. Vascular plexuses were separated by the automated segmentation function software, and the manual correction was performed when the macular structure was intact to ensure appropriate segmentation. All images were carefully reviewed by professional operators. The superficial retinal capillary plexus (SCP) was defined as the area 3 *μ*m below the internal limiting membrane to 15 *μ*m below the inner plexiform layer. The deep capillary plexus (DCP) was defined as the area from 15 to 70 *μ*m below the IPL [12–14] (Figure 1). The VD was defined as the percentage area of pixels occupied by the vascular flow signal relative to the total scanning area. The VD was measured from the en face of OCT on the fovea and parafovea. Only the qualified images (signal strength index >50) were selected for the analysis.

### 2.2. Statistical Analysis

All data were presented as mean ± deviation (range) when they have a normal distribution, while number (percentage) was used to describe non-normal distribution. The parametric quantitative data were compared between groups using independent *t*-test or analysis of variance (ANOVA), when necessary. The least significant difference (LSD) was used to compare two groups after ANOVA. The nonparametric data were compared using Kruskal–Wallis H test, while the distributions of sex in different groups were conducted by the chi-square test. All data were analyzed using IBM SPSS Statistics version 25.0 (IBM Corp. Armonk, NY, USA).

## 3. Results

### 3.1. Study Demographic Data

A total of 25 eyes from 25 healthy volunteers were recruited in the study. The mean age was 51.0 ± 12.0 years (28–66 years). Among them, 10 were male and 15 were female. A total of 25 patients with bilateral RP were enrolled in the study. Moreover, five eyes in three patients with macular epiretinal membrane, one eye in one patient with macular hole, and 14 eyes in 10 patients (four patients with bilateral eyes, six patients with unilateral eye) with poor images were excluded. Only 30 eyes from 17 patients with RP were evaluated in the study. The mean age of the patients was 50.4 ± 11.5 years (32–60 years). Ten patients were male, and seven were female. We classified them into macular intact or defect groups according to the integrity of the ellipsoid zone in the macular fovea region (Table 1 and Figure 1). If the ellipsoid zone is intact, it belongs to the macular intact group; otherwise, it belongs to the macular defect group. Furthermore, 15 eyes from eight patients with RP belong to the macular defect group, the mean age of the patients was 51.3 ± 12.4 years (32–60 years), and the VA was 1.5 (1.2–0.5) (approximately to 20/1000 Snellen equivalent). Fifteen eyes from nine patients belong to the macular intact group, the mean age of patients was 49.0 ± 10.0 years (37–57 years), the VA was 0.30 (0.30–0) (approximately 20/40 Snellen equivalent) in the macular intact group. There was no significant difference in age within the groups (*F* = 0.065 and *P*=0.937, ANOVA) (Table 1). The sex distribution was not significantly different in all groups (*P*=0.672). VA was better in the macular intact group than in the macular defect group (*P*=0.014).

### 3.2. VD and Central Macular Retinal Thickness (CMT) Measured by OCTA

The measurement of VD through analysis of en face OCTA in patients with RP indicated the loss of vascular signal compared to normal individuals (Table 2 and Figure 1). The VD was calculated in the superficial and deep vascular plexuses by automated software. The macular defect group showed greater vessel loss than the macular intact group, especially in the parafovea region (Table 2). The major difference in VD between the macular intact and macular defect groups was in the DCP, especially in the perifovea region (Table 2). There was no significant difference in SCP between the macular defect and intact groups in either the fovea or parafovea regions (*P*=0.296). The DCP of the perifovea region showed greater loss of vascular capillary in the macular defect group than in the macular intact group (*P* < 0.05); however, there was no significant difference between the two groups in the fovea and nasal regions (*P*=0.296, *P*=0.127). The CMT was found to be lower in patients with RP than in healthy individuals (*P* < 0.05) (Table 2). However, there was no significant difference in the inferior region in healthy individuals and patients with RP (*P*=0.147, *P*=0.453). However, the CMT was not significantly different between the macular intact and macular defect groups (*P* > 0.05) (Table 2).

### 3.3. Correlation between Outer VD and DCP/SCP Ratio

After analysis of the multivariate linear regression, the whole DCP was a dependent variable, and the macular defect and the DCP/SCP ratio in the whole, fovea, and parafovea regions were closely related to the whole DCP (*P* < 0.05) (Table 3). The CRT in the fovea and parafovea regions were not related to whole DCP (*P*=0.186, *P*=0.539).

## 4. Discussion

RP is a broad common inherited disease with photoreceptor degeneration. The present study indicates that the VD decreased in patients with RP, especially in patients with the macular defect. There was a greater loss of vessel plexuses in the DCP when the macular fovea was involved, and the central retinal thickness may not be a more sensitive indicator than the DCP in patients with RP.

A previous study pointed out that retinal perfusion was reduced in patients with RP using the OCTA and FA Doppler ultrasonography [15, 16]. Several studies found that the reduced VD was mainly in the deep and superficial plexuses. Moreover, some researchers found that decreased retinal perfusion mainly occurred in the parafovea region [6]. Microvascular attenuation was reported in patients with RP, and the reason for microvascular attenuation is caused by the regulatory vasoconstriction and the decreased metabolic demand [17, 18]. The impaired retinal blood supply might be an early primary event in RP [13]. However, the parts of the retinal vascular supply that were frequently affected were unclear.

In our study, we classified patients with RP into macular intact or defect groups. The results indicated that there was no significant difference in SCP between the two groups, and the major difference was in the DCP. The loss of outer retinal capillaries in the DCP was the main change in the animal of RP [14]. In our study, we classified them into the macular intact or defect group. The results demonstrated that a greater loss of DCP was found in the macular defect group, and the main position for capillary loss in the DCP was in the parafovea region, not the whole and fovea region. The capillary in the DCP was the main nourishment for the outer retinal layers. The decreased retinal perfusion could cause photoreceptor degeneration [13]. A new hypothesis is that outer retinal degeneration could cause vascular remodeling and attenuation [11]. Thus, a greater loss of capillaries in DCP was observed in the macular defect group. Furthermore, why are capillaries in the DCP vulnerable to loss in patients with RP? A previous study demonstrated that more oxygen diffusion from the choroid into the superficial retina was detected when the outer retinal thickness decreased [19, 20]. The capillaries in the SCP were far from the choroid, but when the photoreceptors degenerated, more oxygen was transferred into the DCP, which may cause the dysfunction of the outer retina, and the photoreceptors were prone to degeneration [13]. Moreover, we found that the DCP/SCP ratio was closely related to the whole DCPVD. It stated that the DCP/SCP ratio may be an early indicator to reflect the vascular condition in patients with RP. However, there were some limitations in the study, including small samples in each group and without the follow up to detect the changes of vessel density with progression of disease. The magnification of OCT may be affected by the axial length [21, 22]; we have not adjusted the scans to account for the magnification of images.

## 5. Conclusions

The OCTA could reflect the microvascular changes in either the superficial or deep plexuses in patients with RP. A greater loss of capillaries in the DCP was easily detected when the macular region was involved in patients with RP. The DCP/SCP ratio may be an important indicator to reflect the severity of RP.

## Figures and Tables

**Table 1:** Demographic information of the study.

	Control	RP		Control vs. intact	Control vs. defect	Defect vs. intact
		Macular defect	Macular intact	*P* value	*P* value	*P* value
Number	25	8	9			
Sex (eye/patients)						
Male	10(10)	8(4)	9(5)	0.674^a^		
Female	15(15)	7(4)	6(4)			
Eyes		15	15			
Age, y	51.0 ± 12.0	51.3 ± 12.4	49.0 ± 10.9	0.736	0.959	0.744
Range	(28–66)	(32–60)	(37–57)			
VA (log MAR)	0	1.5(1.2–0.5)	0.3 (0.32–0)	*P* = 0.112	*P* < 0.001	0.014
	Snellen 20/20	Snellen 20/1000	Snellen 20/40			

VA means vision acuity, *P* < 0.05 was considered as significant, ANONA for age, a for Chi-square test, and the VA analysis in different groups were compared by Kruskal–Wallis H test.

**Table 2:** Vessel density and macular retinal thickness in retinitis pigmentosa patients.

	Control	RP	*P* ^a^ value	RP with macular defect	RP with macular intact	Control vs. intact	Control vs. defect	Defect vs. intact
						*P* ^b^ value	*P* ^b^ value	*P* ^b^ value
SCP								
Whole	53.51 ± 1.92	32.98 ± 3.46	<0.001	31.87 ± 3.16	34.32 ± 3.66	<0.001	<0.001	0.262
Fovea	29.28 ± 2.73	12.19 ± 8.22	<0.001	14.38 ± 5.84	9.56 ± 10.51	<0.001	<0.001	0.359
Parafovea	55.92 ± 2.44	35.60 ± 4.30	<0.001	34.04 ± 3.98	37.16 ± 4.42	<0.001	<0.001	0.275
Tempo	54.53 ± 1.76	36.34 ± 6.89	<0.001	36.52 ± 6.36	36.16 ± 8.14	<0.001	<0.001	0.094
Superior	56.85 ± 2.22	35.82 ± 6.40	<0.001	34.04 ± 7.81	37.60 ± 4.82	<0.001	<0.001	0.411
Nasal	55.18 ± 2.28	36.52 ± 3.14	<0.001	36.02 ± 3.60	37.12 ± 2.76	<0.001	<0.001	0.589
Inferior	56.50 ± 2.39	33.81 ± 9.23	<0.001	30.50 ± 6.72	37.78 ± 10.97	<0.001	<0.001	0.24
DCP								
Whole	57.29 ± 6.63	37.20 ± 8.64	<0.001	36.55 ± 1.94	37.98 ± 13.44	<0.001	<0.001	0.801
Fovea	28.71 ± 5.82	23.64 ± 7.91	0.03	21.26 ± 9.66	26.50 ± 4.56	0.426	0.015	0.296
Parafovea	61.19 ± 2.18	42.31 ± 4.29	<0.001	38.96 ± 1.58	45.66 ± 3.29	<0.001	<0.001	0.003
Tempo	60.20 ± 1.73	41.43 ± 4.78	<0.001	38.34 ± 4.52	44.52 ± 2.70	<0.001	<0.001	0.03
Superior	44.04 ± 5.49	62.34 ± 2.07	<0.001	40.12 ± 4.69	47.96 ± 2.72	<0.001	<0.001	0.012
Nasal	61.22 ± 2.19	41.16 ± 5.72	<0.001	38.73 ± 5.28	44.08 ± 8.23	<0.001	<0.001	0.127
Inferior	61.52 ± 2.26	40.93 ± 6.56	<0.001	36.55 ± 4.67	46.18 ± 4.11	<0.001	<0.001	0.006
CRT								
Fovea	239.93 ± 9.59	196.36 ± 32.11	<0.001	180.50 ± 35.25	215.40 ± 13.90	<0.001	<0.001	0.068
Parafovea	307.31 ± 9.29	247.70 ± 29.56	<0.001	236.40 ± 23.89	259.00 ± 32.80	<0.001	<0.001	0.248
Temporal	296.59 ± 13.02	233.90 ± 30.68	<0.001	217.20 ± 21.74	250.60 ± 30.78	<0.001	<0.001	0.083
Superior	312.00 ± 8.43	246.40 ± 34.68	<0.001	227.20 ± 23.76	265.60 ± 34.92	<0.001	<0.001	0.077
Nasal	310.07 ± 10.30	261.64 ± 33.36	<0.001	263.50 ± 36.51	259.40 ± 33.23	<0.001	<0.001	0.851
Inferior	287.80 ± 76.61	248.55 ± 33.40	0.124	238.83 ± 28.05	260.20 ± 38.64	0.147	0.453	0.315

SCP means superficial capillary plexuses, DCP means deep capillary plexuses, CRT means central retinal thickness, a for independent *t*-test, and b for LSD.

**Table 3:** Univariable multilevel linear regression analysis for whole DCP.

Variable	*β* coefficient	95% CI	*P* value
Fovea CRT	−0.146	−0.181	0.186
Parafovea CRT	0.066	−0.054	0.101
Whole DCP/SCP ratio	0.784	44.826–57.887	<0.001
Fovea DCP/SCP ratio	0.164	0.019–0.068	<0.001
Parafovea DCP/SCP ratio	−0.243	−48.862 to −17.057	<0.001
Macular^a^	0.909	7.55–10.58	<0.001

CI means confidence interval, CRT means central retinal thickness, *P* value <0.05 was considered significant, DCP means deep capillary plexuses, and SCP means superficial capillary plexuses, a macular intact was considered as the reference term.

## Data Availability

The underlying data supporting the results of the study can be obtained upon request to the first author (moses.1988@163.com).
